# Frontal Lobe Atrophy in Depression after Stroke

**DOI:** 10.1155/2013/424769

**Published:** 2013-02-24

**Authors:** W. K. Tang, Y. K. Chen, J. Y. Lu, V. C. T. Mok, Winnie C. W. Chu, Gabor S. Ungvari, K. S. Wong

**Affiliations:** ^1^Department of Psychiatry, Chinese University of Hong Kong, Hong Kong; ^2^Department of Psychiatry, Shatin Hospital, Hong Kong; ^3^Department of Neurology, Dongguan People's Hospital, Dongguan, Guangdong, China; ^4^Department of Medicine and Therapeutics, Chinese University of Hong Kong, Hong Kong; ^5^Department of Imaging & Interventional Radiology, Chinese University of Hong Kong, Hong Kong; ^6^School of Psychiatry & Clinical Neurosciences, University of Western Australia, Perth, Western Australia, Australia; ^7^University of Notre Dame Australia/Marian Centre, Perth, Western Australia, Australia

## Abstract

*Background*. Frontal lobe atrophy (FLA) is associated with late life depression. However, the role that FLA plays in the development of depression after stroke (DAS) remains unknown. This study thus examined the association between FLA and DAS. *Methods*. A convenience sample of 705 Chinese patients with acute ischemic stroke admitted to the acute stroke unit of a university-affiliated regional hospital in Hong Kong participated in the study. A psychiatrist administered the Structural Clinical Interview for DSM-IV to all patients and made a diagnosis of DAS three months after the index stroke. *Results*. Eighty-five (12.1%) patients were diagnosed with DAS. In univariate analysis, the DAS patients were more likely to have severe FLA (14.1% versus 5.6%). Severe FLA remained an independent predictor of DAS in multivariate analysis, with an odds ratio of 2.6 (95% confidence intervals = 1.2–5.9). *Conclusions*. The results suggest that FLA may play a role in the pathogenesis of DAS, which supports the hypothesis that cumulative vascular burden may be important in predicting DAS. Further investigations are needed to clarify the impact of FLA on the clinical presentation, treatment response, and outcome of DAS in stroke survivors.

## 1. Introduction

Depression is the most common psychiatric disorder following stroke. Depression is associated with excessive disability, cognitive impairment, and mortality [1]. The neuroanatomical model of depression after stroke (DAS), a depressive illness in patients with well-established cerebrovascular disease, remains unclear. There is no compelling evidence to support the hypothesis of a close relationship between lesion location and DAS [2], although it has been suggested recently that chronic vascular burden may be an important factor in the development of DAS [3]. 

The frontal lobes are thought to be involved in the pathogenesis of depression [4]. Frontal lobe atrophy (FLA) is associated with late life depression [5] and depression occurring in multiple sclerosis [6]. FLA in stroke can be conceptualised as a sign of vascular damage to brain structures [7], and as such it contributes to the development of vascular depression [8]. To date, no study has found an association between FLA and DAS [9–14]. The aim of this study was thus to reexamine the relationship between FLA and DAS in stroke survivors. 

## 2. Materials and Methods

### 2.1. Participants

A total of 3,219 patients with first-ever or recurrent acute ischemic stroke were admitted to the Acute Stroke Unit of the Prince of Wales Hospital (PWH) between June 2004 and July 2008. The PWH is a university-affiliated general hospital serving a population of 800,000 in Hong Kong. Of the 1,353 patients who received an MRI examination, a convenience sample of 705 (52.1%) was recruited for this study. Compared to the participants, the excluded patients (*n* = 648) were older (69.0 ± 11.4 versus 64.4 ± 11.4, *P* < 0.001), less likely to be female (53.5% versus 59.1%, *P* = 0.038), and more likely to have a higher National Institute of Health Stroke Scale (NIHSS) score (6.9 ± 5.8 versus 4.4 ± 3.4, *P* < 0.001). 

The inclusion criteria for the study were (1) Chinese ethnicity, (2) Cantonese as the primary language, (3) aged 18 or above, (4) well-documented (clinical presentation and CT scan of the brain) first or recurrent acute stroke occurring within the seven days prior to admission, and (5) ability and willingness to give consent. The exclusion criteria were (1) transient ischemic attack, cerebral haemorrhage, subdural haematoma, or subarachnoid haemorrhage, (2) history of a central nervous system disease such as tumour, trauma, hydrocephalus, and Parkinson's disease, (3) aphasia, defined as a score of 2 or more in the best language item of the NIHSS [15], and (4) moderate to severe cognitive impairment, defined as a Mini-Mental State Examination (MMSE) [16] score less than 17. 

The study protocol was approved by the Clinical Research Ethics Committee of the Chinese University of Hong Kong. All participants signed a consent form. 

### 2.2. Collection of Demographic and Clinical Data

A research nurse, who was blind to the psychiatric diagnoses, collected the demographic data (age, sex, and education level in terms of school years) and assessed stroke severity using the NIHSS within two days of admission. A research assistant evaluated all participants with the MMSE and the Lubben Social Network Scale (LSNS) [17] three months after the onset of the index stroke. The LSNS is a composite social network scale that was specifically designed for use in the elderly. It measures the level of social support that patients receive and their social interactions with relatives and friends. The LSNS has been translated into Chinese and validated amongst the Hong Kong elderly [18]. Exposure to recent life events was assessed with the Modified Life Event Scale (MLES) [19, 20].

### 2.3. Assessment of DAS

Three months after the onset of the index stroke, a psychiatrist, who was blind to the participants' radiological data, administered the Chinese version of the Structured Clinical Interview for DSM-IV [21, 22] at a research clinic. The timing of the assessment was chosen to avoid the period of transient emotional adjustment to the disability caused by stroke [23].

### 2.4. Radiological Examination

MRI with diffusion-weighted imaging (DWI) and proton density sequences was performed on each participant with a 1.5-T system (Sonata, Siemens Medical, Erlangen, Germany) within seven days of admission.

DWI spin EPI (TR/TE/excitation = 180/122/4, matrix =  128 × 128, FOV = 230 mm, slice thickness/gap⁡ = 5 mm/1 mm, EPI factor = 90, and acquisition time = 55 seconds) with three orthogonally applied gradients was used with values of 1000 and 500. Axial gradient echo images were acquired as the second sequence, with imaging parameters of TR/TE/excitation = 350/30/2, a flip angle of 30 degrees, slice thickness/gap⁡ = 5 mm/0.5 mm, FOV = 230 mm, matrix = 256 × 256, and acquisition time = 5 minutes and 4 seconds. Axial SE T1 (TR/TE/excitation = 425/14/2, FOV = 230 mm, slice thickness/gap⁡ = 5 mm/0.5 mm, matrix = 256 × 256, and acquisition time = 4 minutes, 28 seconds) and TSE T2 (TR/TE/excitation = 2500/120/1, turbo factor = 15, FOV = 230 mm, slice thickness/gap⁡ = 5 mm/0.5 mm, matrix = 256 × 256, and acquisition time = 1 minute, 39 seconds) images were also acquired.

 A neurologist, who was blind to the psychiatric diagnoses, assessed the MRIs to determine the following.

1. *Brain Atrophy*. Brain atrophy was quantified with a three-point visual cortical atrophy scoring method [24] to rate the severity of cortical atrophy on the T1-weighted images. This method provides two standard images that represent mild and moderate lobar atrophy severity. Severe lobar atrophy was recorded when the extent of atrophy on either side of the lobe was more severe than that on the second standard image. Atrophy in the frontal, parietal, temporal and occipital lobes was rated separately. The intrarater reliability of the lobar atrophy measurement, which was tested on 20 cases, was good (the weighted kappa values for the frontal, parietal, and temporal lobes were 0.90, 0.81, and 0.71, resp.).
2. *Brain Infarcts*. The number and volume of acute infarcts were recorded. The total area of acute infarcts on the DWI was measured with manual outlines. Restricted water diffusion was identified on diffusion-weighted images with *b* values of 1000. The total volume was calculated by multiplying the total area by the sum of the slice thickness and the gap. Intrarater reliability tests were performed on 20 participants, yielding kappa values of 0.96 and 0.94 for the volume and number of infarcts, respectively.
3. *White Matter Hyperintensities (WMHs)*. The severity of WMHs was graded using a 4-point scale [25, 26]. Periventricular white matter (PVWMHs) and deep white matter (DWMHs) hyperintensities were scored on axial proton density images. The intrarater agreement was good for both of the PVWMH (weighted kappa: 0.87) and DWMH ratings (weighted kappa: 0.93).

### 2.5. Statistical Analysis

All statistical tests were performed using SPSS for Windows (Release 14.0, SPSS Inc., Chicago, IL, USA). The demographic and clinical variables (age, sex, education level, and NIHSS and MMSE scores) and radiological characteristics of the participants with DAS were compared with those of the non-DASs group using the *χ* ^2^ test, Student's *t*-test, and Mann-Whitney *U* test, as appropriate. Risk factors with a value of *P* < 0.10 were then analysed via multivariate logistic regression using a forward stepwise selection strategy. If the correlations between any of the putative risk factors were ≥0.50, then additional models were examined to rule out collinearity. The odds ratio of any independent risk factor was interpreted as the risk of subsequent DASs when all other risk factors were held constant. The level of significance was set at 0.05. 

## 3. Results

Eighty-five patients were diagnosed with DAS. The participants' demographic, clinical, and MRI characteristics stratified by DAS status are shown in Table 1.

Severe FLA was more common in the DAS group. Participants with DAS were also more likely to be female and to have a history of depression, a lower level of education, and higher NIHSS and MLES scores but lower MMSE and LSNS scores.

The following variables were entered into the regression model: sex, education, severe FLA, history of depression, and the NIHSS, MMSE, MLES, and LSNS scores. Severe FLA was a significant independent predictor of DAS, with an odds ratio of 2.6 (*P* = 0.016) (Table 2). Severe FLA remained a significant predictor of DAS in additional regression models (data not shown), in which either age was entered into the regression (odds ratio = 3.00, *P* = 0.007) or patients with prior depression were excluded (odds ratio = 2.5, *P* = 0.026).

## 4. Discussion 

To the best of our knowledge, this is the first MRI study to show that severe FLA is an independent predictor of DAS.

Although a number of studies have examined the relationship between brain atrophy and DAS, none has focused on the frontal lobes. In addition, only broad measures of atrophy, such as global [12], cortical [9, 14], subcortical [9, 14], and central atrophy, [11, 13] or ventricle-to-brain ratio were analysed in these studies [14], which may explain their negative findings.

FLA in stroke can be the result of degeneration and/or ischemic injuries. Brain atrophy is associated with subcortical white matter lesions both in patients with vascular dementia [27] and in healthy controls [28]. FLA in stroke is also associated with white matter lesions and small vessel disease [7]. Our findings support the recent hypothesis that DAS is related to cumulative vascular brain pathology rather than a single infarct [3]. Another indicator of chronic vascular burden, namely, lacunae, is also associated with DAS [3].

One limitation of this study is that it was conducted in a hospital-based sample, and thus its findings may not be applicable to patients treated in other settings. In addition, the exclusion rate was high which might have resulted in a biased sample. Furthermore, the number of cases of DAS with FLA was small and a large number of possible confounding variables were adjusted in the multivariate analysis; hence the findings should be regarded as tentative and need replication. Finally, FLA was only one of the six significant factors in the final regression model.

## 5. Conclusions

In conclusion, the results of this study indicate that there is an association between FLA and DAS. Further investigations are needed to clarify the impact of FLA on the clinical presentation, treatment response, and outcome of DAS in stroke survivors.

## Figures and Tables

**Table 1:** Univariate and multivariate analyses of the clinical and radiological determinants of DAS.

	DAS	No DAS	P*value
	Mean ± SD	Mean ± SD	
*N*	85	620	
Age	66.9 ± 11.6	66.2 ± 11.6	0.632^†^
Female	50 (58.8)	238 (38.4)	<0.001^‡^
Education (years)	4.4 ± 4.7	5.7 ± 4.7	0.005
Previous stroke	23 (27.1)	123 (19.8)	0.123^‡^
NIHSS during index admission	5.1 ± 3.5	4.4 ± 3.4	0.029
MMSE	24.8 ± 3.6	26.1 ± 3.2	0.002
LSNS	25.5 ± 8.4	30.4 ± 8.2	<0.001^†^
MLES	2.3 ± 1.2	1.7 ± 0.8	<0.001
Past history of depression	14 (16.5)	7 (1.1)	<0.001^‡^
Severe brain atrophy			
Frontal lobe	12 (14.1)	35 (5.6)	0.003^‡^
Temporal lobe	6 (7.1)	76 (12.3)	0.161^‡^
Parietal lobe	14 (16.5)	97 (15.6)	0.845^‡^
Occipital lobe	13 (15.3)	95 (15.3)	1.000^‡^
Periventricular hyperintensities	1.2 ± 0.8	1.3 ± 0.9	0.653
Deep white matter hyperintensities	1.1 ± 0.9	1.1 ± 0.8	0.726
Number of acute infarcts	1.0 ± 1.4	1.3 ± 1.1	0.131
Acute infarct volume (mm^3^)	2.4 ± 5.2	2.9 ± 9.1	0.772

NIHSS: National Institute of Health Stroke Scale; MMSE: Mini-Mental State Examination; LSNS: Lubben Social Network Scale; MLES: Modified Life Event Scale; SD: standard deviation.

*In comparison to the DAS group by means of Mann-Whitney *U* test unless otherwise specified.

^†^In comparison to the DAS group by means of *t*-test.

^‡^In comparison to the DAS group by means of Chi-square *U* test.

**Table 2:** Multivariate logistic model of the clinical and radiological determinants of DAS.

Variable	Odds ratio (95% CI)	*P* value
Severe frontal lobe atrophy	2.648 (1.198–5.856)	0.016
History of depression	11.99 (3.953–36.372)	<0.001
MLES	1.925 (1.490–2.487)	<0.001
LSNS	0.943 (0.915–0.972)	<0.001
NIHSS	1.106 (1.032 –1.185)	0.004
Female	2.312 (1.370–3.902)	0.002
MMSE	—	NS
Education	—	NS

*R* ^2^ = 0.259.

NIHSS: National Institute of Health Stroke Scale; MMSE: Mini-Mental State Examination; LSNS: Lubben Social Network Scale; MLES: Modified Life Event Scale; NS: nonsignificant.
